# Diagnostic Insights and Treatment Approaches for Dermatophytosis Affecting Vellus Hair

**DOI:** 10.1155/drp/3373022

**Published:** 2025-09-18

**Authors:** Rungsima Kiratiwongwan, Charussri Leeyaphan, Pattriya Jirawattanadon, Lalita Matthapan, Waranyoo Prasong, Chatisa Panyawong, Sumanas Bunyaratavej

**Affiliations:** Department of Dermatology, Faculty of Medicine Siriraj Hospital, Mahidol University, Bangkok, Thailand

**Keywords:** dermatomycoses, therapeutics, tinea, tinea of vellus hair, treatment outcome

## Abstract

**Background:** Tinea of vellus hair is a rare condition that is recalcitrant to treatment. It is typically caused by nonanthropophilic dermatophytes. Extant data on this disease remain scarce.

**Aims/Objectives:** This study aimed to delineate the clinical features and treatment outcomes of patients with tinea of vellus hair and to compare the characteristics of patients infected by anthropophilic and nonanthropophilic species.

**Methods:** A 10-year retrospective study was conducted at the Department of Dermatology in a tertiary hospital in Thailand. The study included all patients with tinea of glabrous skin involving vellus hair. Baseline characteristics, clinical data, and treatment outcomes were analyzed.

**Results:** Of the 31 patients in the study, two-thirds of the patients (69%) had a history of using topical medications, mainly steroids and antifungals. The face and extremities were the most common locations for lesions with positive vellus hair. There were no significant differences in data between patients infected with anthropophilic and nonanthropophilic species. Most patients received oral antifungals (80.6%). There was no significant difference in the cure rate between patients who were administered oral antifungals and those who solely utilized topical antifungals. Kaplan–Meier analysis demonstrated the overall median duration to achieve a cure was 5 weeks.

**Conclusion:** The diagnosis of tinea of vellus hair should be considered in cases of tinea of the glabrous skin in exposed areas, especially in patients with a history of topical treatments. Nonanthropophilic dermatophytes are the primary causative agents of tinea of vellus hair. Systemic antifungals with prolonged duration are recommended.

## 1. Introduction

Cutaneous dermatophytosis of hairless skin is a prevalent dermatologic condition caused by a specific group of pathogenic fungi. It is typically treated with topical antifungal medications [1–4]. However, systemic antifungal treatment may be necessary in cases involving hair and nails or when the disease is extensive or recalcitrant in patients with tinea of hairless skin [1, 2, 4]. Vellus hairs, which are short, nonpigmented, and lack the medulla and arrector pili muscle, are commonly found in areas of hairless skin. Previous studies have primarily diagnosed tinea of vellus hair through microscopic examination of skin scrapings that demonstrated vellus hair infested with fungal hyphae [1, 5, 6]. This process requires adept microscopists for accurate identification. Although dermatophyte involvement of vellus hairs is rare, it can result in poor responses to topical antifungal therapy [1].

Earlier studies have provided insights into the clinical features of patients with tinea of vellus hair. However, due to the rarity of this disease, these studies were limited to a small number of patients and were mainly conducted in countries located in temperate zones [1, 5, 6]. Furthermore, the majority of tinea of vellus hair cases was caused by nonanthropophilic dermatophytes, specifically zoophilic and geophilic species that are commonly found in animals and soil, respectively [2, 6]. Infections caused by nonanthropophilic dermatophytes can be transmitted to humans, potentially triggering heightened inflammation in cutaneous lesions [7, 8]. Consequently, the present study set out to determine the clinical features, causative organisms, treatment, and treatment outcomes of patients with tinea of vellus hair. Additionally, the study aimed to compare the characteristics of patients infected with anthropophilic and nonanthropophilic species.

## 2. Methods

This retrospective cohort study was conducted at the Department of Dermatology, Faculty of Medicine Siriraj Hospital, Mahidol University, Bangkok, Thailand. The research protocol was authorized by the institutional review board (approval number Si 777/2021). The investigation included patients diagnosed with tinea of glabrous skin with vellus hair involvement based on microscopic examination, characterized by the presence of fungal hyphae and/or spores both within and outside the vellus hair. Cases with dermatophytosis of the scalp, beard, palms, and soles were excluded. The patients were individuals who visited the outpatient unit of the department of dermatology between 2011 and 2021.

For the microscopic examinations, skin samples, including scales and vellus hair, were collected and immersed in a 20% potassium hydroxide solution. Fungal culture was performed by inoculating each sample in Sabouraud dextrose agar, with and without cycloheximide. The cultures were incubated at 27°C and examined every 4 days for 4 weeks. Microorganism identification was based on observing and describing the macroscopic and microscopic characteristics of the colonies. Treatment approaches were determined by the dermatologists responsible for the care of the patients. Repeated microscopic examinations were performed using skin scrapings during follow-up visits every 2 to 4 weeks to assess mycological cure. Complete resolution of rashes (clinical cure) or absence of fungal pathogens in the microscopic examination (mycological cure) indicated a cure. Baseline characteristics, clinical data, treatments, and outcomes of the patients were retrospectively reviewed. Data from patients infected with anthropophilic and nonanthropophilic species were also compared.

### 2.1. Statistical Analysis

Descriptive statistics, such as median, frequency, percentages, and percentiles, were used to describe the baseline characteristics, clinical data, treatments, and outcomes. Fisher's exact test was utilized to test associations between categorical data, while the Mann–Whitney *U* test was employed to analyze differences in quantitative data. Kaplan–Meier analysis was applied to determine the median time to cure. PASW Statistics (version 18, SPSS Inc., Chicago, IL, USA) was used for all statistical analyses.

## 3. Results

**Figure 1:**
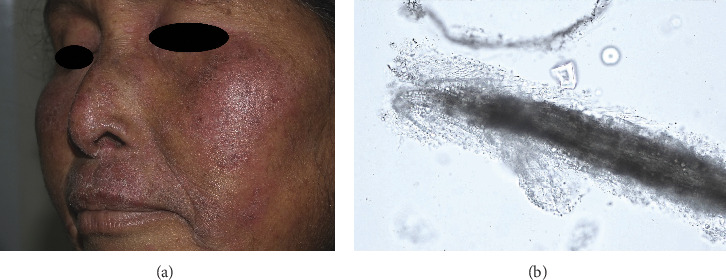
(a) Clinical presentation of a patient afflicted with tinea of vellus hair. (b) Potassium hydroxide examination of a sample from a patient with tinea of vellus hair.

Of the 5807 patients who presented with tinea of glabrous skin at the dermatology clinic from 2011 to 2021, a subset of 31 individuals (0.5%) was diagnosed with tinea of vellus hair.  Figure 1 demonstrates the clinical presentation and potassium hydroxide examination of a patient with tinea of vellus hair.  Table 1 delineates the patients' baseline characteristics, clinical features, treatments, and outcomes. The median age of patients diagnosed with tinea of vellus hair was 48.6 years, with female predominance (80.6%). Fungal species were identified in 19 of these patients. Nonanthropophilic dermatophytes constituted the majority of causative organisms, implicated in 14 out of the 19 cases (73.7%), while anthropophilic dermatophytes were found in the remaining five cases (26.3%). The median age of patients with zoophilic infection tended to be lower than those with the anthropophilic group; however, it was not significantly different. Of the nonanthropophilic dermatophyte cases, 13 (68.4%) were attributed to zoophilic dermatophytes and one (5.3%) to geophilic dermatophytes, with the species identified being *Microsporum canis* (*n* = 8), *Trichophyton mentagrophytes* (*n* = 4), *T. erinacei* (*n* = 1), and *Nannizzia gypsea* (*n* = 1). All five anthropophilic dermatophyte cases were infected with *T. rubrum*.

Regarding treatment, most patients (25/31, 80.6%) were administered oral antifungal medications, whereas six (19.6%) utilized only topical antifungal medications. Of those using topical antifungals, one patient declined oral antifungals; one experienced significant drug interactions with systemic antifungals; and the remaining four were treated exclusively with topical antifungals, adhering to physician preference.

**Figure 2:**
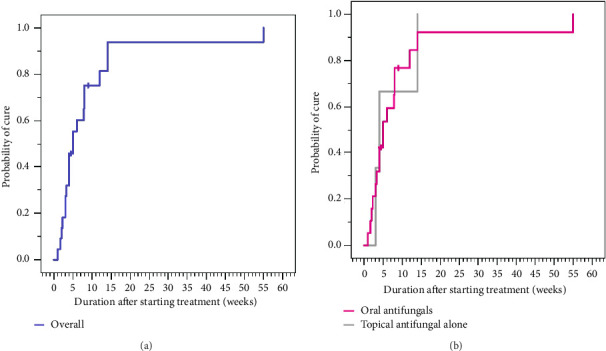
(a) Kaplan–Meier analysis depicting an overall median cure time of 5 weeks. (b) The median time to cure of patients who received oral antifungals and those solely using topical antifungals was 5 weeks and 4 weeks, respectively (*p*=0.964).

Table 1 also compares data between patients infected with anthropophilic and nonanthropophilic species. No significant differences were observed in terms of median age, sex, previous treatments, clinical features, or outcomes between the two groups. Nine patients were lost to follow-up after the initial visit, resulting in an outcome analysis of 22 patients. The overall cure rate and clinical cure rate in the study was 20 out of 22 patients (90.9%; Table 1). Mycological cure data were available for 18 patients, of whom 16 achieved mycological cure (88.9%). All patients treated with terbinafine (*n* = 8), itraconazole (*n* = 4), and griseofulvin (*n* = 1) were cured, whereas 66.7% (4 out of 6 cases) of those treated with fluconazole achieved a cure. There was no significant difference in the cure rate between patients who were administered oral antifungals and those who solely utilized topical antifungals. Kaplan–Meier analysis (Figure 2) demonstrated that after 4, 8, and 12 weeks of treatment, 45.5%, 75.2%, and 81.4% of patients with tinea of vellus hair, respectively, achieved remission. The median duration to achieve a cure in overall was determined to be 5 weeks. The median time to cure did not significantly differ between patients who received oral antifungals and those solely using topical antifungals (5 weeks vs. 4 weeks, respectively; *p*=0.964).

## 4. Discussion

This retrospective study involved the large number of adult patients diagnosed with tinea of vellus hair. The median age of the patients was 48.6 years, and there was a notable female predominance (80.6%). Similar findings have been reported in previous studies, which either found an equal number of male and female patients [2, 6] or a female predominance [1]. However, the median age in our study was notably higher at 48.6 years, compared to 5–28 years observed in the prior studies [1, 2, 6]. This disparity can likely be attributed to the predominant adult demographic seeking care at our Department of Dermatology, as only two children (under 18 years of age) were included in the current study. In this study, lesions indicating fungal invasion in vellus hair were commonly located in exposed areas, notably the face and extremities.

The median duration of rashes in our patient cohort was 10 weeks. Previous studies have also reported varying durations of rashes in patients with tinea of vellus hair [1, 2, 5], with some cases lasting as long as 10 years [2]. The median duration of rash did not show a significant difference between the nonanthropophilic and zoophilic groups. However, a previous study on dermatophytosis of glabrous skin found that patients with anthropophilic infections had significantly longer lesion durations than those with zoophilic infections (12 weeks vs. 4 weeks, respectively) [3]. In our study's nonanthropophilic and anthropophilic groups, most patients had a history of using topical medications, primarily steroids and antifungal drugs. It is known that topical steroids can inhibit the normal cutaneous response to fungal pathogens [9], potentially facilitating fungal invasion of vellus hair. Additionally, when topical steroids are used with topical antifungals, the steroids may suppress the antifungal properties of the medications [9].

Regarding the causative organisms for tinea of vellus hair, the predominant dermatophytes identified in this study were nonanthropophilic, encompassing both zoophilic and geophilic dermatophytes. This observation aligns with preceding reports [2, 6]. One study even demonstrated that all cases of tinea of vellus hair were caused by nonanthropophilic species [1]. This prevalence may be because zoophilic and geophilic species tend to induce more severe inflammation than anthropophilic species [8]. Fungal-specific factors—such as host adaptation, enzyme secretion, production of inflammatory agents and toxins, and release of immunomodulatory substances—influence the degree of inflammation. Species that are less adapted to coexist with humans typically cause highly inflammatory skin infections [10].

In terms of treatment, systemic antifungal medications were prescribed in nearly all cases of tinea of vellus hair in the current investigation. This approach is consistent with several previous observational studies [1, 5, 6], where systemic antifungals were uniformly administered to patients, resulting in the resolution of lesions or a mycological cure in every case. Our research revealed an overall cure rate of 90.9%, with no significant difference in rates between the nonanthropophilic (80%) and anthropophilic groups (100%). In Sun et al.'s study [2], the vast majority of patients (87.5%) received systemic antifungals (terbinafine or griseofulvin), while the remaining 12.5% received only topical antifungals. The mycological cure rate in that study was reported to be 43.8%.

The study by Sun and colleagues also reported treatment outcomes in patients infected with tinea of vellus hair [2]. Different patient proportions achieved varying treatment success across three metrics: mycological cure rate, improvement without isolating the fungal pathogen, and clinical sign resolution with positive or no culture. Specifically, the mycological cure rate was higher in nonanthropophilic cases (55.6%) than in anthropophilic cases (28.6%). In contrast, anthropophilic cases had a higher rate of improvement without isolating the fungal pathogen (42.8%) compared to nonanthropophilic cases (22.2%). Finally, cases with a resolution of clinical signs, with either a positive culture or an unperformed culture, were relatively similar at 14.3% (anthropophilic) and 11.1% (nonanthropophilic). The variance in treatment outcomes could be attributed to the variation in fungal species present within each study.

The present study found no significant difference in cure rates and median time to cure between patients treated with oral antifungals and those using topical treatments alone. This may be due to the low prevalence of tinea of vellus hair, particularly among patients treated solely with topical agents, which limited the statistical power for comparison. Since systemic antifungal therapy was used in nearly all cases of tinea of vellus hair in both our study and previous reports—resulting in lesion resolution or mycological cure in most cases—oral antifungal medication is recommended as the first-line treatment for vellus hair tinea. However, a well-designed study with a larger cohort of patients is warranted.

Kaplan–Meier analysis in our study revealed that after 4, 8, and 12 weeks of treatment, 45.5%, 75.2%, and 81.4% of patients with tinea of vellus hair were in remission, respectively. The median time to cure was 5 weeks. Based on these findings, the authors recommend prolonged duration of treatment to achieve disease resolution.

### 4.1. Limitations

In this retrospective study, tinea of vellus hair was diagnosed in only 31 out of the 5807 patients (0.5%) who had tinea of glabrous skin. The low prevalence of tinea of vellus hair led to insufficient statistical power for patient comparisons. Additionally, there was some missing data due to the study's retrospective nature. Moreover, this study did not determine whether the fungal elements represented endothrix or ectothrix invasion. Dermoscopy may provide diagnostic insights in cases of tinea of vellus hair, such as scaly plaques, translucent hairs, follicular pustules, and broken hairs [6]. However, such data were not available for our cases.

## 5. Conclusions

Tinea of vellus hair is a rare condition, as indicated by the low prevalence of 0.5% in this study. Clinicians should consider the diagnosis of tinea of vellus hair in cases of tinea affecting the glabrous skin in exposed areas, especially in patients with a history of previous topical treatments such as antifungals and corticosteroids. The primary causative agents of tinea of vellus hair are nonanthropophilic dermatophytes, particularly zoophilic species. To achieve resolution of the disease, the authors recommend the use of oral antifungal medications as the mainstay of treatment and emphasize the importance of a prolonged treatment duration.

## Figures and Tables

**Table 1:** Characteristics of patients diagnosed with tinea of vellus hair (*n* = 31).

	Number (%)			*p*
	All cases (*n* = 31)	Cases with identified fungal culture (*n* = 19)		
		NAD^†^ (*n* = 14)	AD^‡^ (*n* = 5)	
Age (years), median (P25, P75)	48.6 (25.5, 57.2)	48.6 (36.5, 59.1)	29.4 (22.1, 64.6)	0.579
Female	25 (80.6)	12 (85.7)	3 (60)	0.272
Previous treatments^†^ (*n* = 29)				
Topical treatments	20 (69.0)	11/14 (78.6)	2/3 (66.7)	1.000
Topical steroids	14 (48.3)	10/14 (71.4)	2/3 (66.7)	1.000
Topical antifungals	10 (34.5)	6/14 (42.9)	1/3 (33.3)	1.000
Topical antibiotics	1 (3.4)	1/14 (7.1)	0/3 (0)	1.000
Topical unidentified OTC	5 (17.2)	0/14 (0)	0/3 (0)	1.000
Systemic steroid	7 (24.1)	3/14 (21.4)	2/3 (66.7)	0.191
Systemic antifungal	5 (16.1)	3/14 (21.4)	0/3 (0)	1.000
Duration of symptoms (weeks), median (P25, P75)	10 (3, 24)	5 (2, 24)	4 (2.1, 22)	0.774
History of contact with pets (*n* = 21)	16 (76.2)	9/11 (81.8)	1/2 (50.0)	0.423
Area of lesions				
Confined in one area	18 (58.1)	9 (64.3)	2 (40.0)	0.603
More than one area/concomitant other type(s) of dermatophytosis	13 (41.9)	5 (35.7)	3 (60.0)	
Locations of positive vellus hair^‡^				
Face	10 (32.2)	5 (35.7)	2 (40)	1.000
Arm and forearm	9 (29)	5 (35.7)	0 (0)	0.257
Thigh and leg	9 (29)	3 (21.4)	2 (40)	0.570
Groin	4 (12.9)	1 (7.1)	1 (20)	0.468
Trunk	1 (3.2)	1 (7.1)	0 (0)	1.000
Treatment				
Oral antifungals	25 (80.6)	11 (78.6)	4 (80)	1.000
Fluconazole	10 (32.3)	5 (35.7)	1 (20)	
Terbinafine	8 (25.8)	4 (28.6)	2 (40)	
Itraconazole	6 (19.4)	2 (14.3)	0 (0)	
Griseofulvin	1 (3.2)	0 (0)	1 (20)	
Only topical antifungals	6 (19.4)	3 (21.4)	1 (20)	
Cure rate (*n* = 22)	20 (90.9)	8/10 (80)	5/5 (100)	0.524
Clinical cure (*n* = 22)	20 (90.9)	8/10 (80)	5/5 (100)	0.524
Mycological cure (*n* = 18)	16 (88.9)	8/10 (80)	2/2 (100)	1.000

*Note:* OTC, over-the-counter medication.

Abbreviations: AD, anthropophilic dermatophytes; NAD, nonanthropophilic dermatophytes.

^†^One patient may have one or more previous treatments.

^‡^One patient may have one or more locations.

## Data Availability

The data that support the findings of this study are available from the corresponding author upon reasonable request.

## References

[1] Gómez-Moyano E., Crespo-Erchiga V. (2010). Tinea of Vellus Hair: An Indication for Systemic Antifungal Therapy. *British Journal of Dermatology*.

[2] Sun P. L., Lin Y. C., Wu Y. H., Kao P. H., Ju Y. M., Fan Y. C. (2018). Tinea folliculorum Complicating Tinea of the Glabrous Skin: An Important yet Neglected Entity. *Medical Mycology*.

[3] Bunyaratavej S., Kiratiwongwan R., Komoltri C., Lertrujiwanit K., Leeyaphan C. (2022). Predictive Equation to Identify Infection Due to Anthropophilic or Zoophilic Dermatophytes Based on Clinical Features and Risk Factors: A Ten-Year Retrospective Study. *Indian Journal of Dermatology, Venereology and Leprology*.

[4] Pires C. A., Cruz N. F., Lobato A. M., Sousa P. O., Carneiro F. R., Mendes A. M. (2014). Clinical, Epidemiological, and Therapeutic Profile of Dermatophytosis. *Anais Brasileiros de Dermatologia*.

[5] Atzori L., Aste N., Aste N., Pau M. (2012). Tinea faciei Due to Microsporum Canis in Children: a Survey of 46 Cases in the District of Cagliari (Italy). *Pediatric Dermatology*.

[6] Gómez-Moyano E., Crespo Erchiga V., Martínez Pilar L. (2016). Using Dermoscopy to Detect Tinea of Vellus Hair. *British Journal of Dermatology*.

[7] Bassiri-Jahromi S. (2013). Epidemiological Trends in Zoophilic and Geophilic Fungi in Iran. *Clinical and Experimental Dermatology*.

[8] Weitzman I., Summerbell R. C. (1995). The Dermatophytes. *Clinical Microbiology Reviews*.

[9] Dhaher S. (2020). Tinea Incognito: Clinical Perspectives of a New Imitator. *Dermatology Reports*.

[10] Hube B., Hay R., Brasch J., Veraldi S., Schaller M. (2015). Dermatomycoses and Inflammation: the Adaptive Balance Between Growth, Damage, and Survival. *Journal de Mycologie Médicale*.

